# Understanding Parental Perspectives on Childhood Hearing Impairment and Timely Interventions

**DOI:** 10.7759/cureus.78025

**Published:** 2025-01-26

**Authors:** Nada Alharbi, Daniyah Baqalaqil, Hams Alharthi, Nouf Almalki, Samar Altoukhi, Nawaf Alzahrani, Abdullah Sanedi, Farees Almohaimeed, Abdulrahman AlOtaibi, Hosam Amoodi

**Affiliations:** 1 College of Medicine, University of Jeddah, Jeddah, SAU; 2 College of Medicine, Albaha University, Albaha, SAU; 3 College of Medicine, Faculty of Medicine, King Abdulaziz University, Jeddah, SAU; 4 College of Medicine, Qassim University, Buraydah, SAU; 5 College of Medicine, Department of Colorectal Surgery, University of Jeddah, Jeddah, SAU; 6 Department of Colorectal Surgery, Dr. Soliman Fakeeh Hospital, Jeddah, SAU; 7 College of Medicine, Department of Otolaryngology - Head and Neck Surgery, University of Jeddah, Jeddah, SAU

**Keywords:** childhood hearing loss, cross sectional study, developmental delay, parental awareness, questionnaire, saudi arabia

## Abstract

Objectives: Hearing impairment during childhood is a widespread health issue. Prompt recognition and timely intervention are vital for the advancement of language skills. Insufficient parental knowledge can lead to a delay in diagnosing and treating a condition, which can have a negative impact on academic performance. The goals of our study were to assess the knowledge levels of parents regarding childhood hearing loss and to evaluate their attitudes toward the availability of hearing services.

Methods: A cross-sectional study was conducted using an online questionnaire. Participants, totaling 339, were recruited from various regions across Saudi Arabia through a stratified random sampling technique to ensure diverse representation. Recruitment was conducted via social media platforms, including Twitter, and WhatsApp. The data were examined using Microsoft Excel (Microsoft® Corp., Redmond, WA) and Statistical Product and Service Solutions (SPSS, version 26.0; IBM SPSS Statistics for Windows, Armonk, NY).

Result: This study consisted of 339 participants, of whom 295 (87.0%) were female. The 41-50 age group was the most represented at 35.7% (121/339). A total of 76.1% of the participants demonstrated inadequate knowledge regarding childhood hearing loss and its connection to developmental delay. Although the participants had limited knowledge, a significant majority of 82.9% expressed a favorable disposition toward hearing services.

Conclusions: This study emphasizes the existence of significant gaps in knowledge and diverse attitudes toward childhood hearing loss among caregivers. This underscores the urgent need for public health initiatives to tackle these gaps through educational and awareness campaigns. By promoting a well-informed and optimistic approach to hearing health, we can improve the results of early detection and intervention, thereby reducing the long-term effects on speech, language, and cognitive development.

## Introduction

Hearing impairment during childhood is a widespread and significant health issue worldwide [1]. Furthermore, it is widely acknowledged as a prominent source of anguish for both the child and their parents. Hearing loss can manifest as either a congenital or acquired impairment, both of which have detrimental effects on various aspects of child development, including cognition, emotions, social interactions, and, most notably, speech and language acquisition [2]. According to The Diagnostic and Statistical Manual of Mental Disorders, Fifth Edition (DSM-5), developmental delay refers to significant delays in the development of intellectual and adaptive functioning, typically identified in children under the age of five, when full assessments may not yet be possible. These delays can be in multiple areas, including cognitive, motor, and speech development. Hence, it is imperative for parents to have a comprehensive understanding of audiology assessments and hearing services in order to identify, address, and mitigate any potential detrimental health occurrences [3]. According to the World Health Organization (WHO), out of the 466 million people with hearing loss, 34 million are children who are unable to hear normally [4]. To determine how common hearing loss is in children, researchers in Saudi Arabia studied 10,000 of them. The results showed that 127 (1.27%) of the children had left-sided hearing loss, 226 (2.26%) had right-sided hearing loss, and 947 (9.47%) had bilateral hearing loss [5]. Sixty percent of hearing loss cases in children younger than 15 years old are caused by preventable causes, such as overexposure to loud noise, secondhand smoke, and audio device use [6]. A study conducted in Qassim, Saudi Arabia, in 2020 included 243 parents as participants [7]. This study assessed their knowledge about childhood hearing loss, including its causes, risk factors, and the importance of early detection and intervention. The findings revealed that 103 participants (42.4%) demonstrated a high level of knowledge regarding these topics, while 140 participants (57.6%) exhibited a low level of knowledge. Despite this knowledge gap, the majority of parents (224, or 92.2%) expressed a favorable perception of audiology services, indicating their general support for these services. These results underscore the need for targeted educational interventions to enhance parental understanding of childhood hearing loss and its management. In a separate study conducted in Taif, Saudi Arabia, which included 308 parents as participants [8], it was found that only 15 individuals (5%) had adequate knowledge regarding childhood hearing loss. Children with hearing impairment frequently encounter difficulties in their linguistic progression and academic achievements [9,10]. Identifying hearing impairment in children at an early stage and promptly providing intervention can greatly benefit their language development [11]. Our review of the literature revealed that, while previous studies have examined parental knowledge and attitudes toward childhood hearing loss in Saudi Arabia, our research addresses a unique gap by focusing specifically on awareness of hearing loss and attitudes toward interventions and hearing services. Unlike prior studies, which often concentrated on specific regions or smaller populations, our study utilizes stratified random sampling to include diverse participants from across Saudi Arabia, ensuring comprehensive representation. This broader focus on parental understanding and service accessibility provides valuable insights for shaping effective public health strategies, with the aim of improving awareness and promoting early intervention for childhood hearing loss nationwide.

## Materials and methods

Study design, inclusion criteria, and sample size

This cross-sectional study was conducted using an online questionnaire targeting parents from various regions across Saudi Arabia. A total of 339 participants were recruited through a stratified random sampling technique to ensure diverse representation. Participants were selected by dividing the population into strata based on geographic regions across Saudi Arabia. Proportional random sampling was then applied within each stratum to ensure a balanced representation of parents from different regions.

The inclusion criteria required participants to be parents with children of any age, capable of communicating in either English or Arabic, the study languages, and willing to provide informed consent. Individuals who resided outside of Saudi Arabia, faced language barriers not addressed by the translations, worked in the healthcare sector (to minimize professional bias and ensure the sample represented general parental knowledge), or were unable to provide informed consent were excluded from participation.

The sample size for this study was determined using the formula provided by Raosoft, Inc. (Seattle, WA). The formula considers the desired 95% confidence interval, a standard deviation of 1.96, and a margin of error of 5%. Hence, the necessary sample size for a population of 20,000 was determined to be 377.

Questionnaire and scoring system

A structured, pre-designed questionnaire was adapted from a previous study by Ahmed Elrefaie et al. [12], with modifications made to fit the context of this study. Permission to use and modify the questionnaire was obtained from the author. It was available in both English and Arabic to accommodate participants' language preferences. Participants were given the option to complete the survey in their preferred language, and accommodations, such as translations, were provided for those with limited proficiency in either language. The survey was distributed through various social media platforms, including Twitter, and WhatsApp, between November 2023 and January 2024, ensuring broad dissemination and accessibility to different demographics across Saudi Arabia. The questionnaire consisted of 44 questions divided into three sections. The first section gathered sociodemographic information, including age, gender, educational level, income, marital status, and geographic region, to capture the diversity of participants. The second section assessed participants’ knowledge of childhood hearing loss, with 28 questions addressing causes, risk factors, prevention, and interventions. These questions were designed to evaluate both general and specific knowledge, such as awareness of the link between jaundice, head trauma, and hearing loss. Scoring was based on assigning 1 point for each correct answer and 0 for incorrect answers, with cumulative scores categorized as good, moderate, or low levels of knowledge based on Bloom's cut-off point. The third section evaluated attitudes toward hearing loss and hearing services, using five questions aimed at assessing participants’ openness to interventions such as newborn hearing screening and their perception of hearing services.

In the attitude section, participants were asked five questions about their views on hearing impairment, with responses categorized as "yes," "no", or "I don’t know". To quantify these responses, a score of 2 was assigned for a "yes" answer (indicating a positive attitude) and 0 for "no" (indicating a negative attitude). For responses of "I don’t know," a score of 1 was assigned. This was done to capture uncertainty or lack of opinion, distinguishing it from clear positive or negative attitudes. The total score for the attitude section could range from 0 to 10. Based on the final score, participants' attitudes were classified into three categories: positive, neutral, or negative.

Respondents who achieved a score of 8 or higher were classified as having a high level of positive attitude, whereas those who scored 7 displayed a neutral attitude. Any value less than 7 was in the negative attitude range.

This questionnaire was validated through a pilot study conducted on 15 participants. Their feedback was incorporated, and adjustments were made, followed by another pilot study to ensure clarity and comprehensiveness [12].

Ethical approval

The study was approved by the Institutional Review Board (IRB) at the University of Jeddah (approval number: UJ-REC-275). All participants provided informed consent before participating in the survey.

Statistical analysis

The analysis was conducted using Statistical Product and Service Solutions (SPSS, version 26.0; IBM SPSS Statistics for Windows, Armonk, NY). Sociodemographic characteristics, along with measurements of knowledge and attitude, were investigated, utilizing descriptive statistics to demonstrate the study results. A chi-square test was used to assess the distribution of participant levels of knowledge and attitude across different sociodemographical subgroups. A p-value of less than 0.05 was deemed significant.

## Results

A total of 475 responses have been received through an online survey. Three hundred thirty-nine of them were eligible with the research criteria; this is because 70 were excluded (67 for working in the healthcare sector and three for reporting inconsistent data) and 66 neither have any children nor are primary caregivers. As illustrated in Table 1, the sociodemographic characteristics of participants indicate a higher proportion of female participants, with 295 respondents (87.0%), compared to male participants, who were fewer, at 44 respondents (13.0%). The age group of 41-50 years had the highest representation with 121 individuals (35.7%), while the 20-30 years age group was the least represented with only 42 individuals (12.4%). The majority of the participants were Saudi nationals, encompassing 324 individuals (95.6%), and a small fraction were non-Saudi, making up to 15 participants (4.4%). Examining the social status, a vast majority of participants were married, accounting for 301 of the responses (88.8%), with singles being the least represented at only four (1.2%). With respect to educational level, most participants held a bachelor's degree, totaling 190 individuals (56.0%), and the least number had postgraduate degrees, with 32 participants (9.4%). Regarding monthly income, the largest group of participants (a total of 117) earned less than 5,000 SAR (34.5%), whereas those earning more than 15,000 SAR were the least, with 58 participants (17.1%). Lastly, when looking at the regional distribution, the western region had the highest number of participants at 234 (69.0%), whereas the northern region had the least, with only 13 individuals (3.8%).

**Table 1:** Sociodemographic characteristics of 339 eligible participants Exclusions were made for healthcare workers (67), inconsistent data (3), and individuals without children or caregiving roles (66). The majority were female (87%), Saudi nationals (95.6%), married (88.8%), and had a bachelor's degree (56%). The largest age group was 41-50 years (35.7%), and the western region had the highest participation (69%).

Item		N	N%
Gender	Male	44	13.0%
	Female	295	87.0%
Age	20-30	42	12.4%
	31-40	110	32.4%
	41-50	121	35.7%
	51-65	66	19.5%
Nationality	Saudi	324	95.6%
	Non-Saudi	15	4.4%
Social status	Single	4	1.2%
	Married	301	88.8%
	Divorced	24	7.1%
	Widow	10	2.9%
Educational level	Elementary	5	1.5%
	Middle School	7	2.1%
	High School Diploma	68	20.1%
	Diploma	37	10.9%
	Bachelor's Degree	190	56.0%
	Postgraduate Degree	32	9.4%
Monthly income	Less than 5000 SAR	117	34.5%
	5000-10,000 SAR	84	24.8%
	10,000-15,000 SAR	80	23.6%
	More than 15,000 SAR	58	17.1%
Region	Eastern Region	30	8.8%
	Western Region	234	69.0%
	Southern Region	41	12.1%
	Northern Region	13	3.8%
	Central Region	21	6.2%

Referring to Table 2, participant responses regarding audiology concerns and information seeking were mixed. A total of 134 participants reported having previously visited an audiologist (39.5%), while a larger group (199 in total) indicated that they had not (58.7%). Only a minimal number (six respondents) were unsure or did not know if they had ever visited an audiologist (1.8%).

**Table 2:** Participant responses regarding audiology visits and concerns about childhood hearing Specifically, 39.5% of participants had visited an audiologist, 58.7% had not, and 1.8% were unsure. Regarding concerns about their child's hearing and the need for more information, 49.6% expressed concern and a desire for more information, 43.4% did not share this concern, and 7.1% were uncertain.

Item	Yes, N (N%)	No, N (N%)	I don’t know, N (N%)
Previous visit to an audiologist?	134 (39.5%)	199 (58.7%)	6 (1.8%)
Are you worried about your child's hearing and need more information?	168 (49.6%)	147 (43.4%)	24 (7.1%)

When asked about concerns regarding their child's hearing and the need for more information, a slight majority of the respondents (a total of 168) acknowledged their worry and desire for additional information (49.6%). On the other hand, 147 participants did not share this concern (43.4%), and 24 participants were uncertain or lacked enough information to decide (7.1%).

The classification of the participants' knowledge regarding childhood hearing loss, as demonstrated in Table 3, was skewed towards the lower end, with a majority of 258 individuals (76.1%) demonstrating low knowledge. Meanwhile, 54 participants (15.9%) displayed moderate knowledge, and only a minority of 27 (8.0%) were classified as having good knowledge. Descriptive analysis revealed a mean of 13.67 scores ± 5.479 SD.

**Table 3:** Classification of the participants knowledge and attitude toward childhood hearing loss A majority (76.1%) demonstrated low knowledge, with only 8.0% showing good knowledge. The mean knowledge score was 13.31 ± 4.28 SD. In terms of attitude, 82.9% held a positive attitude, with a mean attitude score of 8.27 ± 1.71 SD.

Scale		N	N%	Mean	Standard Deviation
Knowledge	Good	27	8.0%	13.31	4.28
	Moderate	54	15.9%	19	1
	Low	258	76.1%	11	4
Attitude	Positive	281	82.9%	8.27	1.71
	Neutral	34	10.0%	6	1
	Negative	24	7.1%	3	2

In terms of attitude toward childhood hearing loss, a mean of 8.73 scores ± 1.965 SD were reported among the study sample. Despite the predominant lack of in-depth knowledge, attitudes toward childhood hearing loss were predominantly positive. A notable 281 of the respondents (82.9%) reported having a positive attitude. A neutral stance was reported by 34 participants (10.0%), and a minority of 24 participants (7.1%) held a negative attitude towards childhood hearing loss.

In Table 4, participants' knowledge of causes and risk factors for hearing loss in children was mixed. A significant number were aware that jaundice can cause hearing loss, with 234 (69%) affirming this, while a similar number recognized that delayed crying at birth can lead to hearing loss (174, 51.3%). Over half of the participants knew that measles can cause hearing loss (170, 50.1%), as well as the potential impact of chemicals/medications (171, 50.4%). Other recognized causes included low birth weight (157, 46.3%) and maternal infection and medication during pregnancy (158, 46.6%).

**Table 4:** Parents' knowledge of causes and risk factors of loss of hearing among children Many recognized jaundice (69%) and delayed crying at birth (51.3%) as causes, but fewer acknowledged noise exposure (29.8%) or head trauma (25.7%). Awareness of risk factors such as family history (26.5%) and consanguineous marriages (31%) was limited. Knowledge of treatment options and preventative measures, such as breastfeeding and vaccinations, was also low.

Item	Yes, N (N%)	No, N (N%)	I don’t know, N (N%)
Knowledge regarding causes of hearing loss			
Babies may be born with a lost sense of hearing.	38 (11.2%)	278 (82%)	23 (6.8%)
Central nervous system infection can cause hearing loss in children.	115 (33.9%)	212 (62.5%)	12 (3.5%)
Convulsions can cause hearing loss in children.	136 (40.1%)	179 (52.8%)	24 (7.1%)
High fever can cause hearing loss in children.	88 (26%)	231 (68.1%)	20 (5.9%)
Newborn infections can cause hearing loss in children.	154 (45.4%)	136 (40.1%)	49 (14.5%)
Measles can cause hearing loss in children.	170 (50.1%)	115 (33.9%)	54 (15.9%)
Maternal infection and medication during pregnancy can cause hearing loss in children.	158 (46.6%)	144 (42.5%)	37 (10.9%)
Chemicals/medications can cause hearing loss in children.	171 (50.4%)	125 (36.9%)	43 (12.7%)
Jaundice can cause hearing loss in children.	234 (69%)	55 (16.2%)	50 (14.7%)
Delayed crying at birth can lead to hearing loss.	174 (51.3%)	72 (21.2%)	93 (27.4%)
Low birth weight can lead to hearing loss in babies.	157 (46.3%)	31 (9.1%)	151 (44.5%)
Congenital malformations of the head can lead to hearing loss in children.	122 (36%)	187 (55.2%)	30 (8.8%)
Head trauma can lead to hearing loss in children.	87 (25.7%)	219 (64.6%)	33 (9.7%)
Exposure to noise can lead to hearing loss in children.	101 (29.8%)	167 (49.3%)	71 (20.9%)
Ear secretions and otitis media can lead to hearing loss.	84 (24.8%)	217 (64%)	38 (11.2%)
Repeated upper respiratory infections can lead to otitis media.	117 (34.5%)	195 (57.5%)	27 (8%)
The fumes (tobacco/fires) can cause otitis media.	155 (45.7%)	107 (31.6%)	77 (22.7%)
Children with hearing loss can have similar educational opportunities as their hearing peers.	57 (16.8%)	261 (77%)	21 (6.2%)
Treatment for hearing loss is available.	76 (22.4%)	240 (70.8%)	23 (6.8%)
Knowledge regarding risk factors and preventives of hearing loss			
Family history can be a risk factor for hearing loss in children.	90 (26.5%)	221 (65.2%)	28 (8.3%)
Consanguineous marriage can be a risk factor for hearing loss in children.	105 (31%)	167 (49.3%)	67 (19.8%)
Breastfeeding for the first 6 months may reduce/prevent otitis media.	101 (29.8%)	209 (61.7%)	29 (8.6%)
Routine vaccinations for children can reduce middle ear infections.	113 (33.3%)	185 (54.6%)	41 (12.1%)
Is it possible to diagnose impairment and hearing loss in children immediately after birth?	89 (26.3%)	170 (50.1%)	80 (23.6%)
Children affected by hearing loss and impairment can go to school.	54 (15.9%)	267 (78.8%)	18 (5.3%)
Delayed acquisition of communication skills (speaking/language) can be a sign of hearing loss and impairment in children.	64 (18.9%)	244 (72%)	31 (9.1%)

On the other hand, less than a third of the participants acknowledged that exposure to noise (29.8%) and head trauma (25.7%) can lead to hearing loss in children. Only 57 participants (16.8%) believed that children with hearing loss can have similar educational opportunities as their hearing peers. Additionally, knowledge about treatment availability for hearing loss was relatively low, with 76 (22.4%) affirming its availability. Moreover, exclusively 84 (24.8%) people acknowledged that ear secretions and otitis media are related to hearing loss.

Furthermore, In Table 4, participants' knowledge regarding risk factors for hearing loss in children was assessed. It was found that a majority were not aware that family history could be a risk factor for hearing loss, with 221 participants (65.2%) not acknowledging this factor and only 90 (26.5%) affirming it. Similarly, consanguineous marriages were not widely recognized as a risk factor, with 167 respondents (49.3%) unaware of the association, although 105 participants (31%) identified this as a risk factor. Breastfeeding for the first six months as a preventative measure against otitis media was also not well recognized, with only 101 individuals (29.8%) agreeing that it could reduce or prevent such infections, and a majority of 209 (61.7%) disagreed. When it comes to routine vaccinations' ability to reduce middle ear infections, a greater number (113, 33.3%) acknowledged this benefit, yet a significant number (185, 54.6%) did not.

The possibility of diagnosing hearing impairment and loss in children immediately after birth was not commonly recognized, with only 89 (26.3%) affirming this and a substantial 170 (50.1%) unaware of the possibility. Regarding educational opportunities for children affected by hearing loss, a low number (54, 15.9%) believed that these children could attend school, while a high number (267, 78.8%) did not. Lastly, there was a lack of awareness concerning the significance of delayed acquisition of communication skills as an indicator of hearing loss and impairment, with only 64 participants (18.9%) aware of this link, whereas a majority of 244 (72%) did not recognize this sign.

**Figure 1:**
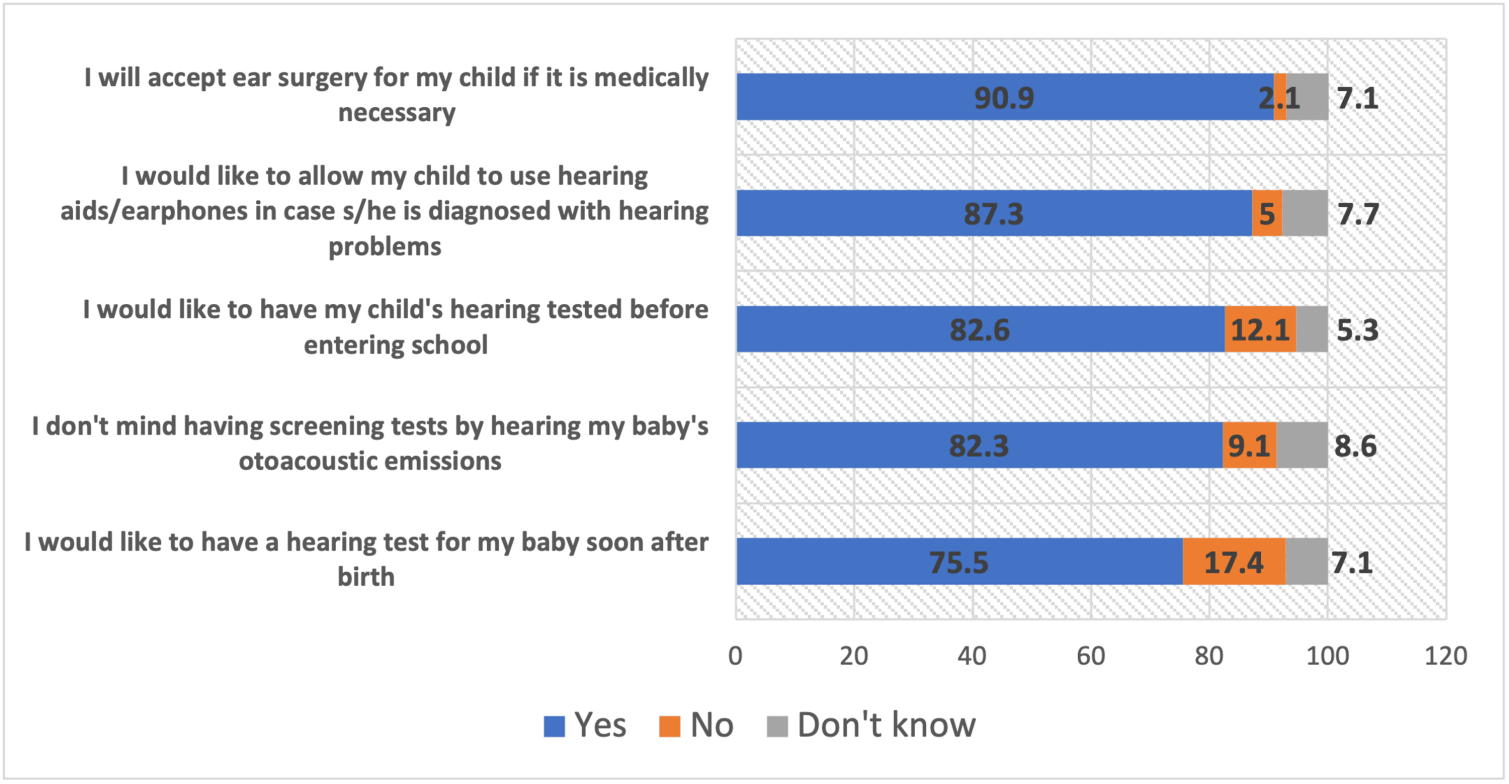
Parents' attitude toward loss of hearing among children This figure illustrates parents' favorable attitudes toward early hearing assessment and interventions. A majority expressed interest in newborn hearing tests (75.5%), supported otoacoustic emissions screening (82.3%), and preferred hearing tests before school (82.6%). Additionally, most parents were open to hearing aids (87.3%) and ear surgery if medically necessary (90.9%).

As presented in Figure 1, parents showed a favorable attitude towards early hearing assessment and interventions for their children. A significant majority of parents (256 in total) expressed a desire to have a hearing test for their baby soon after birth (75.5%). In addition, a substantial number of respondents (a total of 279) did not object to having screening tests by hearing their baby’s otoacoustic emissions (82.3%). Moreover, there was a strong inclination towards preventative health measures, with 280 parents wishing to have their child’s hearing tested before starting school (82.6%). When considering the use of hearing aids, an overwhelming majority, accounting for 296 parents, were agreeable to allowing their child to use hearing aids/earphones if diagnosed with hearing problems (87.3%). Regarding medical interventions, an impressive 308 parents stated they would accept ear surgery for their child if it was deemed medically necessary (90.9%).

Table 5 presents the association between participants' sociodemographic characteristics and their knowledge and attitudes toward childhood hearing loss. The analysis revealed a significant association between the participants' region of residence and their knowledge of the subject, with the western region showing a notably higher proportion of participants with low knowledge (182), resulting in a P-value of 0.047 (p<0.05), indicating statistical significance. Other sociodemographic factors, such as gender, age, nationality, social status, educational level, and monthly income, did not demonstrate a statistically significant association with the participants' knowledge or attitudes toward childhood hearing loss.

**Table 5:** Association between participants' knowledge and attitude with sociodemographic characteristics of participants A chi-squire test was used to assess the distribution of participant levels of knowledge and attitude across different sociodemographic subgroups. A significant association was found between region of residence and knowledge, with the western region having a higher proportion of participants with low knowledge (p=0.047). No other sociodemographic factors, including gender, age, nationality, social status, educational level, or income, showed a significant association. *(p<0.05) indicating statistical significance

Variable			Knowledge					Attitude			
		Good	Moderate	Low	X^2 ^value	P value	Positive	Neutral	Negative	X^2 ^value	P-value
Gender	Male	1	8	35	2.296	0.317	34	5	5	1.593	0.451
	Female	26	46	223			247	29	19		
Age	20-30	3	8	31	8.217	0.223	39	3	0	6.462	0.373
	31-40	11	12	87			87	13	10		
	41-50	12	23	86			99	11	11		
	51-65	1	11	54			56	7	3		
Nationality	Saudi	25	52	247	0.650	0.722	269	33	22	1.060	0.589
	Non-Saudi	2	2	11			12	1	2		
Social status	Single	0	1	3	10.764	0.096	4	0	0	2.277	0.893
	Married	23	44	234			249	31	21		
	Divorced	3	4	17			19	3	2		
	Widow	1	5	4			9	0	1		
Educational level	Elementary	0	0	5	7.185	0.708	4	0	1	11.831	0.297
	Middle School	0	1	6			6	0	1		
	High School	4	9	55			62	6	0		
	Diploma	3	8	26			31	2	4		
	Bachelor's Degree	17	34	139			153	21	16		
	Postgraduate Degree	3	2	27			25	5	2		
Monthly income	Less than 5000	7	19	91	2.506	0.868	101	11	5	8.076	0.233
	5000-10,000	9	12	63			70	9	5		
	10,000-15,000	7	15	58			63	11	6		
	More than 15,000	4	8	46			47	3	8		
Region	Eastern Region	3	7	20	15.676	0.047*	27	2	1	6.554	0.585
	Western Region	15	37	182			191	24	19		
	Southern Region	8	3	30			33	6	2		
	Northern Region	1	1	11			10	1	2		
	Central Region	0	6	15			20	1	0		

## Discussion

Early childhood hearing loss is detrimental to socioemotional development, speech, language, and learning [2]. In addition, it causes stigmatization, which may result in a permanent adverse effect on individuals by promoting parental denial of the illness [13]. This study aimed to assess the knowledge and attitudes of parents and caregivers toward childhood hearing loss, drawing upon a comprehensive survey that garnered 339 eligible responses. The results illuminated significant disparities in knowledge levels and attitudes, emphasizing the need for targeted educational interventions and policy reforms.

Knowledge and awareness disparities

The predominance of low knowledge levels about childhood hearing loss among our respondents, with 76.1% demonstrating low knowledge, echoes findings from similar studies, including the referenced study from the western region of Saudi Arabia, which also reported considerable gaps in parental knowledge [7]. This commonality underscores a widespread issue across varied demographics and geographical locations, pointing to a universal need for enhanced educational programs aimed at raising awareness about childhood hearing loss causes, risk factors, and the critical importance of early detection and intervention. It is also valid to note that 69% of the participants in this study were from the western region of Saudi Arabia, which may have contributed to the similarity in the overall findings.

A striking 76.1% of participants demonstrated low knowledge regarding childhood hearing loss, a figure that notably diverges from previous research, indicating higher levels of awareness in other contexts of Saudi Arabia [7,14]. This discrepancy might be attributed to variations in sociodemographic profiles and the scope of educational outreach across studies.

Moreover, in the analysis of the parents’ knowledge, individuals in this study had fair information regarding chemicals/medications and their ability to cause hearing loss in children, as well as jaundice, delayed crying at birth, and low birth weight. This contrasts with findings from Alsudays et al. [7], despite similarities in overall rates.

Attitudes toward childhood hearing loss

Our findings reveal a nuanced spectrum of attitudes toward childhood hearing loss, with nearly half of the participants holding a positive outlook. This is comparable to findings from a similar study conducted in Qassim, Saudi Arabia, which also highlighted a generally positive disposition among parents toward audiological services, despite prevalent knowledge gaps [7]. However, such attitudes provide a fertile ground for implementing effective awareness and intervention programs, suggesting that, while knowledge may be lacking, the willingness to engage with and seek out hearing health services is present among a significant portion of the population. Notably, the Qassim study reported better knowledge levels; 42.4% possessed good knowledge, while 57.6% possessed poor knowledge, despite the large similarity of characteristics between the samples. This observation could be due to different methodological approaches, as this study allocated a cut-off point of 80% for the classification of good knowledge, whereas the other study’s cut-off point was 50%. This suggests more comparable findings. Overall, most studies conducted in Saudi Arabia and neighboring countries such as the United Arab Emirates have demonstrated a similar skew toward positive attitudes [1-5]. The discrepancy between knowledge and attitude or expectations may result in negative psychological reactions when a related condition arises. This is evident as an Indian study illustrated that parents unaware of hearing loss were less likely to recognize the possibility of it being the cause of their child’s delayed speech production and response, which may ultimately affect the lives of both parents and children dramatically [15].

Sociodemographic Influences

The study identified no significant association between sociodemographic factors, such as gender, age, nationality, social status, educational level, and monthly income with knowledge and attitudes toward childhood hearing loss. This diverges from some aspects of the literature, which suggest that socioeconomic status and educational levels can influence health knowledge and attitudes. Furthermore, a study conducted in Sharjah, United Arab Emirates, reported a significant association between knowledge levels, educational level, and age [16]. However, it aligns with findings from a similar study, where major knowledge gaps were not exclusively tied to specific demographic factors [17]. This suggests that misinformation and lack of awareness about childhood hearing loss are pervasive issues that cut across socioeconomic boundaries, reinforcing the need for universally accessible educational initiatives.

Differences with previous studies

Our study found similar gaps in parental knowledge about childhood hearing loss as studies in Saudi Arabia and other regions. For example, Al-Yahya et al. and Alsudays et al. [2,7] also highlighted low knowledge levels among parents, particularly regarding the causes, risks, and importance of early detection. Similarly, studies from Aljuaid et al. and Elrefaie et al. found positive attitudes toward interventions despite limited knowledge, which mirrors our findings [8,12]. Studies such as Alshehri et al. and Bedaiwi et al. also showed limited awareness in Jeddah and Madinah, reinforcing the need for public health campaigns to address knowledge gaps and promote early intervention [13,14]. However, our study adds a new dimension by employing a stratified random sampling technique across various regions of Saudi Arabia, ensuring a more diverse and comprehensive representation of parental knowledge and attitudes. This broader approach allows us to highlight region-specific needs and emphasize the importance of tailored educational initiatives to improve early diagnosis and intervention across the country.

Implications for public health policy and practice

The collective findings from this and similar studies articulate a clear call to action for public health policymakers and practitioners. There is an evident need for comprehensive, accessible, and culturally sensitive public health campaigns aimed at demystifying childhood hearing loss. Such campaigns should prioritize the dissemination of information on early signs of hearing loss, the importance of neonatal screening, and the pathways to access care and intervention services. The effective implementation of early hearing detection and intervention (EHDI) programs depends heavily on parental attitudes and understanding regarding hearing loss, particularly in developing nations where there have been concerns expressed about culturally based ignorance and hostility toward childhood disability [18].

## Conclusions

This study aimed to assess parental knowledge and attitudes toward childhood hearing loss in Saudi Arabia, and it revealed significant gaps in both areas. The findings underscore the importance of addressing these gaps through public health initiatives focused on education and awareness. By improving parental understanding and fostering a more positive outlook on hearing health, early detection and intervention for childhood hearing loss can be enhanced, leading to better speech, language, and cognitive development outcomes.

While this study provides valuable insights, its limitations must be acknowledged. The reliance on self-reported data, the small sample size, and the potential for selection bias inherent in online survey methodologies could affect the generalizability of the findings. As a result, the conclusions drawn from this study may not be applicable to the broader population. Future research should aim to employ mixed-method approaches to explore the complexities of parental knowledge and attitudes toward childhood hearing loss more deeply. Additionally, longitudinal studies assessing the impact of targeted educational interventions on these parameters would be valuable for informing public health strategies.

## Appendices

**Table 6:** Questionnaire The structured, pre-designed questionnaire used in this study was adapted from a previous study by Elrefaie et al. [12]. Modifications were made to the original instrument, and it was used as a study tool after obtaining the necessary permission from the original publisher.

Consent
By clicking on the ‘‘Agree’’ button, you will have granted the researchers the full right to use your answers for research purposes only. Do you agree to participate?
Agree
Disagree
Work field
Do you work in the healthcare field?
Yes
No
Sociodemographic characteristics of participants
Gender
Male
Female
Age
___
Nationality
Saudi
Non-Saudi
Social status
Single
Married
Divorced
Widow
Educational level
I don’t have a degree
Elementary
Middle school
High school diploma
Diploma
Bachelor's degree
Postgraduate degree
Monthly income
Lees than 5000
5000-10,000
10,000-15,000
More than 15,000
Region
Eastern Region
Western Region
Southern Region
Northern Region
Central Region
Do you have any children?
Yes
No
Are you a caregiver or guardian of a child/children?
Yes
No
Parents knowledge of causes, risk factors, prevention, and factors associated with hearing loss in children
Babies may be born with a lost sense of hearing.
Yes
No
I don’t know
Central nervous system infection can cause hearing loss in children.
Yes
No
I don’t know
Convulsions can cause hearing loss in children.
Yes
No
I don’t know
High fever can cause hearing loss in children.
Yes
No
I don’t know
Newborn infections can cause hearing loss in children.
Yes
No
I don’t know
Measles can cause hearing loss in children.
Yes
No
I don’t know
Maternal infection and medication during pregnancy can cause hearing loss in children.
Yes
No
I don’t know
Chemicals/medications can cause hearing loss in children.
Yes
No
I don’t know
Jaundice can cause hearing loss in children.
Yes
No
I don’t know
Delayed crying at birth can lead to hearing loss.
Yes
No
I don’t know
Low birth weight can lead to hearing loss in babies.
Yes
No
I don’t know
Congenital malformations of the head can lead to hearing loss in children.
Yes
No
I don’t know
Head trauma can lead to hearing loss in children.
Yes
No
I don’t know
Exposure to noise can lead to hearing loss in children.
Yes
No
I don’t know
Ear secretions and otitis media can lead to hearing loss.
Yes
No
I don’t know
Repeated upper respiratory infections can lead to otitis media.
Yes
No
I don’t know
The fumes (tobacco/fires) can cause otitis media.
Yes
No
I don’t know
Family history can be a risk factor for hearing loss in children.
Yes
No
I don’t know
Consanguineous marriage can be a risk factor for hearing loss in children.
Yes
No
I don’t know
Breastfeeding for the ﬁrst 6 months may reduce/prevent otitis media.
Yes
No
I don’t know
Routine vaccinations for children can reduce middle ear infections.
Yes
No
I don’t know
Is it possible to diagnose impairment and hearing loss in children immediately after birth?
Yes
No
I don’t know
Children affected by hearing loss and impairment can go to school.
Yes
No
I don’t know
Delayed acquisition of communication skills (speaking/language) can be a sign of hearing loss and impairment in children.
Yes
No
I don’t know
Children with hearing loss can have similar educational opportunities as their hearing peers.
Yes
No
I don’t know
Treatment for hearing loss is available.
Yes
No
I don’t know
Participants' attitude towards screening and management of hearing loss in children.
Do you agree or disagree with the following statement?
Previous visit to an audiologist
Yes
No
I don’t know
I would like to have a hearing test for my baby soon after birth.
Yes
No
I don’t know
I don't mind having screening tests by hearing my baby's otoacoustic emissions.
Yes
No
I don’t know
I would like to have my child's hearing tested before entering school.
Yes
No
I don’t know
I would like to allow my child to use hearing aids/earphones in case s/he is diagnosed with hearing problems.
Yes
No
I don’t know
I will accept ear surgery for my child if it is medically necessary.
Yes
No
I don’t know
Are you worried about your child's hearing and need more information?
Yes
No
I don’t know
